# Silver Nanoparticle–Silk Protein Nanocomposites: A Synergistic Biomimetic Approach for Advanced Antimicrobial Applications

**DOI:** 10.3390/biomimetics10100669

**Published:** 2025-10-05

**Authors:** Mauro Pollini, Fabiana D’Urso, Francesco Broccolo, Federica Paladini

**Affiliations:** Department of Experimental Medicine, University of Salento, Via Monteroni, 73100 Lecce, Italy; fabiana.durso@unisalento.it (F.D.); francesco.broccolo@unisalento.it (F.B.)

**Keywords:** silver nanoparticles, silk proteins, antimicrobial nanocomposites, wound healing, biomedical applications, green synthesis

## Abstract

The escalating global crisis of antimicrobial resistance demands innovative therapeutic strategies that transcend conventional approaches. This comprehensive review examines the groundbreaking synergistic integration of silver nanoparticles (AgNPs) with silk proteins (fibroin and sericin from *Bombyx mori*) to create advanced nanocomposite materials for biomedical applications. While extensive literature exists for AgNPs and silk proteins individually, a limited number of studies have explored their synergistic combination. This review consolidates this fragmented knowledge to establish the foundational framework for an emerging field. The unique properties of silk proteins as natural reducing, stabilizing, and capping agents enable environmentally friendly AgNPs synthesis while creating intelligent therapeutic platforms with emergent properties. These hybrid materials demonstrate superior performance in terms of antimicrobial efficacy, biocompatibility, and accelerated wound healing compared to the individual components. The nanocomposites exhibit broad-spectrum activity against multidrug-resistant pathogens while maintaining exceptional biocompatibility and promoting tissue regeneration. This integration represents a promising evolution toward biomimetic therapeutic platforms that work in harmony with biological systems, offering sustainable solutions to contemporary healthcare challenges.

## 1. Introduction

The escalating global crisis of antimicrobial resistance (AMR) has created an unprecedented urgency for innovative therapeutic strategies that overcome the limitations of conventional approaches. With multidrug-resistant infections claiming over 700,000 lives annually and projections indicating 10 million deaths by 2050 if current trends continue, the biomedical community faces a critical issue that demands ground-breaking solutions [1,2].

The scientific literature presents a remarkable dichotomy in antimicrobial biomaterial research. Based on the data provided by the Web of Science Core Collection, more than 54.000 documents are related to silver NPs, comprising 10% of the total published documents in the field of nanomaterials, reporting their broad-spectrum antimicrobial efficacy, multiple mechanisms of action, and diverse applications across healthcare domains [3]. Silver nanoparticles (AgNPs), in particular, unlike conventional antibiotics that target specific cellular processes, exert antimicrobial effects through multiple pathways, significantly reducing the likelihood of resistance development [4,5].

Concurrently, silk proteins, namely fibroin and sericin derived from *Bombyx mori* silkworm cocoons, have established themselves as premier biocompatible materials, supported by an increasing number of publications demonstrating their exceptional wound healing properties, biodegradability, and tissue regenerative capabilities [6,7].

Beyond *Bombyx mori,* which dominates the biomedical use, several non-mulberry silks such as *Antheraea pernyi* and *Samia/Philosamia ricini* offer additional opportunities for biomaterial development, due to their distinct amino acid profiles and secondary-structure signatures, which determine different mechanical behavior and biodegradability [8,9]. In this context, pairing AgNPs with non-mulberry silk matrices has recently delivered antimicrobial functionality alongside modified wetting/thermal behavior, thus broadening the design space for release control and scaffold mechanics [10].

*Bombyx mori* silk fibroin, the structural protein comprising 70–75% of silk weight, provides remarkable mechanical strength through its hierarchical β-sheet crystalline structure, while sericin, historically considered textile waste, has emerged as a valuable bioactive agent with intrinsic antimicrobial, antioxidant, and wound healing properties [11,12,13,14]. Beyond their classical roles as structural (fibroin) and adhesive (sericin) proteins, silk biopolymers provide a versatile chemical platform with amino, carboxyl, and hydroxyl functionalities that can mediate both the reduction of Ag^+^ to Ag^0^ and the stabilization of nascent silver nanoparticles. This dual function enables residue-minimizing, “green” syntheses and facilitates intimate interfacial coupling between AgNPs and silk matrices [15]. The convergence of silver nanotechnology with silk protein engineering represents more than a simple combination of materials; it embodies a valuable option toward biomimetic synergism where the integrated system exhibits emergent properties that surpass the sum of individual contributions. This approach aligns with nature’s own strategies, where biological systems achieve optimal performance through sophisticated integration of multiple functional elements in different multiscale structures [16].

Despite this wealth of individual component research, the systematic exploration of their synergistic integration represents an underexplored frontier in antimicrobial biomaterials. This could represent a significant scientific opportunity for explaining underdeveloped promising applications and for providing evidence of their remarkable therapeutic potential. The limited number of publications focusing on AgNP–silk protein combinations suggests that while individual components have been extensively studied, their synergistic potential has not been fully recognized or systematically explored. This gap presents both a significant scientific opportunity for advancing understanding of biomimetic approaches and a practical challenge in terms of standardization and reproducibility of synthesis protocols [17].

Recent pioneering studies have begun to unveil the remarkable potential of this synergistic approach. Silk proteins serve as natural reducing, stabilizing, and capping agents for AgNPs synthesis, enabling environmentally friendly production while enhancing biocompatibility [15,18]. Moreover, emerging evidence suggests that molecular interactions between silver ions and silk protein functional groups create unique microenvironments that amplify antimicrobial efficacy while maintaining excellent tissue compatibility.

This comprehensive review addresses critical knowledge gaps by providing systematic synthesis of AgNP–silk protein synergistic research, establishing a foundational knowledge framework for this emerging field. The literature discussed in this review was identified through a comprehensive, non-systematic search of Web of Science, Scopus, and PubMed, using combinations of keywords, such as “silver nanoparticles”, “silk fibroin”, “sericin”, “antimicrobial nanocomposites”, “wound healing”, “green synthesis”. Peer-reviewed articles published between 2020 and 2025 were considered, and reference lists of key papers were screened to capture additional relevant studies.

Our analysis reveals that the integration creates multifunctional therapeutic platforms that simultaneously address infection control, wound healing acceleration, and tissue regeneration. The evidence demonstrates that AgNP–silk protein nanocomposites represent not merely an incremental advancement, but a transformative platform technology that could redefine standards of care in antimicrobial therapy and regenerative medicine.

## 2. Silk Proteins: Structural Foundation for Biomedical Innovation

Silk fibroin (SF), comprising approximately 70–75% of silk fiber weight, represents one of nature’s most sophisticated structural proteins. The primary structure consists of heavy chain polypeptides (H-fibroin, 390 kDa) and light chain polypeptides (L-fibroin, 26 kDa) connected via a single disulfide bond at the C-terminus, forming stable H-L complexes that are further associated with glycoprotein P25 (30 kDa) in a 6:6:1 ratio [19,20,21]. The hierarchical organization of fibroin creates its remarkable properties through a sophisticated multi-scale architecture. The primary structure of the heavy chain is the key for the structural and biological roles of silk fibroin, being characterized by a complex aminoacidic composition and patterning, where the four most represented amino acids are glycine (Gly, 46%), alanine (Ala, 30%), serine (Ser, 12%), and tyrosine (Tyr, 5%) [22]. Silk fibroin (SF) exhibits a hierarchical structure where β-sheet crystallites are organized into nanofibrils within an amorphous matrix. This nano-fibrillar architecture is responsible for SF’s exceptional strength-to-density ratio, as the rigid β-sheet crystallites provide mechanical strength while the flexible amorphous matrix contributes extensibility, elasticity, and toughness. The alternating flexible and crystallizable regions create conformational protein polymorphism, enhanced by high glycine content that enables secondary structure transitions between α-helices and β-sheets due to its low steric hindrance [22].

For biomedical applications, fibroin can be processed into diverse formats including films, hydrogels, nanofibers, microspheres, and three-dimensional scaffolds [23]. The remarkable versatility processing enables tunable material properties; for example, specific treatments induce β-sheet formation, creating water-insoluble materials with enhanced mechanical properties, while controlled pH and ionic strength can modulate gelation kinetics and final material characteristics [24,25]. The exceptional biocompatibility of fibroin is related to well-characterized molecular pathways that promote tissue regeneration and healing. Fibroin activates multiple cellular signaling cascades including phosphoinositide 3-kinase (PI3K/AKT), mitogen-activated protein kinase (MEK1), and c-Jun N-terminal kinase (JNK) pathways, resulting in enhanced cell migration, proliferation, and survival [23,26]. Moreover, the biodegradability of silk proteins represents a crucial advantage for biomedical applications. Proteolytic enzymes degrade silk proteins in vivo, with degradation rates controllable through processing conditions, crosslinking strategies, and molecular weight modulation. This controlled degradation enables sustained therapeutic agent release while allowing gradual replacement by natural tissue [16].

Sericin, historically discarded as textile waste (50,000 tonnes annually), has emerged as a valuable biomedical resource with unique properties complementary to fibroin [12,27]. This hydrophilic protein acts as a natural adhesive, binding fibroin filaments together while contributing elasticity to the silk fibers. With molecular weights ranging from 20–310 kDa depending on extraction conditions, sericin exhibits remarkable compositional diversity that directly influences its bioactivity [28]. Sericin’s therapeutic potential stems from its rich amino acid composition, particularly the abundance of hydrophilic residues including serine (30%), aspartic acid, and glycine [29]. The bioactivity of sericin encompasses remarkable therapeutic properties that extend far beyond simple biocompatibility. Indeed, beyond biocompatibility and biodegradability characteristics, sericin’s multifunctional nature include anti-inflammatory, antimicrobial, antioxidant, and photoprotective activities [30,31]. In particular, the anti-inflammatory properties of sericin stem from its sophisticated ability to regulate inflammatory mediator release, particularly through the modulation of key cytokines including interleukin-1 (IL-1) and tumor necrosis factor-alpha (TNF-α), which serve as critical orchestrators of inflammatory responses.

The antimicrobial efficacy of sericin derives particularly from cysteine content, which contributes to bacterial inhibition through distinctive molecular mechanisms. The sulfhydryl groups present in cysteine residues facilitate the formation of weak hydrogen bonds with oxygen and nitrogen atoms in bacterial systems, generating reactive compounds that effectively disrupt essential enzymatic and metabolic processes critical for microbial survival and proliferation. Sericin’s antioxidant capabilities represent another crucial aspect of its bioactivity profile, manifesting through efficient reactive oxygen species (ROS) scavenging mechanisms that help maintain cellular redox balance. The protein’s high concentrations of serine and threonine residues enable effective chelation of transition metal ions, which are key catalysts in oxidative reactions [13,31]. In addition, recent work highlights that sericin itself, when acting as reducing/stabilizing agent for AgNPs, retains and even amplifies antioxidant, anti-inflammatory, antidiabetic, and antibacterial functions, strengthening its role as an active player in the composite rather than a passive binder [32].

## 3. Silver Nanoparticles: Antimicrobial Mechanisms and Strategic Optimization

Silver nanoparticles have emerged as one of the most promising antimicrobial agents in modern biomedical applications, exerting broad-spectrum antimicrobial effects through multiple, simultaneous mechanisms that collectively minimize the likelihood of resistance development [33]. The comprehensive review by Rodrigues et al. has provided crucial insights into these antimicrobial mechanisms, demonstrating how silver nanoparticles operate through complex, interconnected pathways that make bacterial adaptation exceptionally difficult [34].

The primary antimicrobial mechanism of silver nanoparticles involves the controlled release of silver ions (Ag^+^) from nanoparticle surfaces, creating a dynamic system where therapeutic activity is sustained over extended periods. Upon bacterial contact, AgNPs undergo surface oxidation processes that facilitate ion release, with the liberated Ag^+^ ions exhibiting remarkable affinity for sulfur-containing biomolecules that are essential for bacterial survival [35]. Silver ions demonstrate exceptional ability to penetrate bacterial cell walls and membranes, subsequently binding to critical intracellular targets through multiple interaction pathways. The DNA interaction mechanism involves silver ions intercalating between DNA base pairs, disrupting both replication and transcription processes essential for bacterial survival and reproduction [35,36,37]. Simultaneously, protein denaturation occurs through silver binding to cysteine residues in essential proteins, causing conformational changes that result in complete functional loss of critical bacterial enzymes and structural proteins. The interference with cellular energetics represents another crucial antimicrobial mechanism, where silver ions disrupt respiratory chain enzymes, particularly those containing sulfur-rich cofactors essential for electron transport and ATP synthesis [35,36,37]. This metabolic disruption is compounded by membrane disruption effects, where silver ions alter membrane permeability and electrical potential, leading to uncontrolled leakage of cellular contents and ultimately bacterial death [37,38]. The combination of these mechanisms creates a comprehensive assault on bacterial viability that operates across multiple cellular systems simultaneously.

The oxidative stress component represents a secondary but equally important antimicrobial mechanism that amplifies the direct effects of silver ions [38]. When AgNPs interact with bacterial cells, they trigger the generation of reactive oxygen species (ROS) through multiple pathways that target essential cellular components. Silver ions can catalyze the formation of ROS by interacting with molecular oxygen and cellular reducing agents, leading to the production of superoxide anions, hydroxyl radicals, and hydrogen peroxide. These ROS cause extensive cellular damage through lipid peroxidation, compromising membrane integrity and creating additional pathways for silver ion penetration. Moreover, protein oxidation and DNA strand breaks create a comprehensive oxidative assault that compounds the direct ionic effects, with genetic lesions that prevent bacterial replication and repair processes [38,39,40]. The size-dependent surface reactivity of AgNPs influences the rate of ROS production, with smaller nanoparticles exhibiting higher catalytic activity due to increased surface area and greater proportion of surface atoms. While the exact mechanisms of ROS production may vary depending on nanoparticle characteristics and cellular environment, the consistent observation of oxidative stress across different bacterial species confirms the universal nature of this antimicrobial pathway [41,42,43].

**Figure 1:**
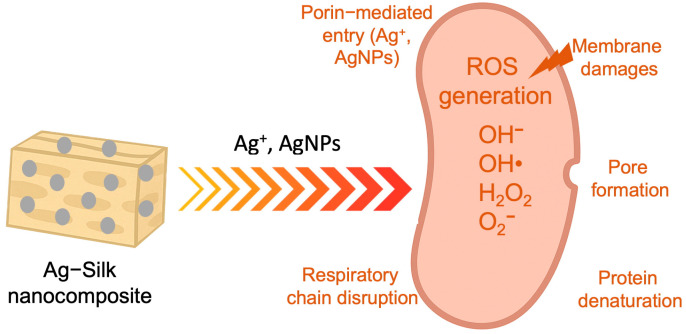
Key antimicrobial mechanisms of silver ions and silver nanoparticles.

The simultaneous attack on multiple cellular targets makes it virtually impossible for bacteria to develop effective resistance mechanisms, as they would need to simultaneously adapt to both ionic silver toxicity and intense oxidative stress. Figure 1 shows the key antimicrobial mechanisms associated with silver ions and silver nanoparticles in the development of nanocomposite-based materials for biomedical applications.

The antimicrobial efficacy of silver nanoparticles depends critically on physicochemical parameters that influence ion release kinetics, cellular uptake efficiency, and overall biological interactions. Recent research by Bruna et al. has provided comprehensive insights into how particle characteristics directly impact antimicrobial performance, demonstrating that careful optimization of these parameters can dramatically enhance therapeutic efficacy while minimizing potential adverse effects [39].

Particle size emerges as perhaps the most critical parameter influencing antimicrobial performance through multiple interconnected mechanisms. Smaller nanoparticles in the 1–10 nm range provide dramatically larger surface area-to-volume ratios, which directly translates to enhanced ion release rates and more rapid achievement of bactericidal concentrations. The enhanced cellular penetration capability of sub-10 nm particles enables them to traverse bacterial cell walls more efficiently, delivering antimicrobial activity directly to intracellular targets where it can exert maximum effect [37]. Research has consistently shown that optimal antimicrobial activity typically occurs with AgNPs dimensions remaining within 50 nanometers, with the most effective range identified between 10 and 15 nanometers, where enhanced stability, superior biocompatibility, and maximum antimicrobial potency are simultaneously observed [44,45,46]. Nanoparticles within the 5–10 nanometer dimensional range manifest dual properties, exerting both bacteriostatic and bactericidal effects against various bacterial strains, including those resistant to conventional antibiotics [44,45,46]. The mechanism underlying this size–efficacy relationship involves direct interaction of nanoparticles with bacterial cell membranes. Smaller particles facilitate adhesion to cellular surfaces and provoke significant alterations in lipid bilayer structure, resulting in increased membrane permeability, structural damage, and cell death. This process is amplified when smaller-sized nanoparticles are employed [44,47].

The high surface-to-volume ratio characteristic of smaller nanoparticles, combined with specific crystallographic surface structures, constitutes the determining factor for their superior antibacterial activity [44,47].

The findings by Stabryla et al. demonstrate that crystal facet architecture plays a crucial role in determining particle–microbe interactions and subsequent biological effects. While surface area may contribute to antimicrobial activity in certain morphologies, a comprehensive investigation examining the bactericidal efficacy of geometrically distinct silver nanoparticles against *Escherichia coli* as a representative Gram-negative pathogen revealed more complex relationships. The study employed cubic, disc-shaped, and pseudospherical geometries, evaluating their antimicrobial potency through systematic dose–response analysis. The experimental results demonstrated that different particle shapes produced markedly disparate antimicrobial outcomes, with cubic nanoparticles achieving complete bacterial inactivation while disc-shaped and pseudospherical variants resulted in less than 25% pathogen elimination [48]. As reported by Pal et al., it may be speculated that silver nanoparticles with the same surface areas but with different shapes have different effective surface areas in terms of active facets; in particular, the reactivity of silver is promoted by high-atom-density facets such as {111}, which provides direct interaction with the bacterial surface [46]. The effect of NP shape on antibacterial activity is currently highlighted in the literature. Specifically, since spherical NPs can efficiently release silver ions, they are widely employed in antibacterial research compared to their counterparts. Additionally, the sharp edges and vertices of triangular NPs enable enhanced interactions with bacterial membranes, facilitating better penetration and bacteria disruption [3].

The comprehensive understanding of silver nanoparticle mechanisms and optimization strategies provides the foundation for rational design of advanced antimicrobial systems. When combined with the unique properties of silk proteins, these insights enable the development of synergistic materials that address the complex challenges of modern antimicrobial therapy while offering unprecedented opportunities for clinical innovation and improved patient outcomes.

## 4. Synthesis Strategies for AgNP–Silk Protein Integration

The synthesis of silver nanoparticles using silk proteins as both reducing and stabilizing agents represents a fundamental progress toward environmentally sustainable nanotechnology, eliminating the need for toxic chemicals traditionally required in nanoparticle synthesis while simultaneously creating materials with superior biocompatibility and enhanced therapeutic performance [49].

Moreover, the formation of silver nanoparticles through in situ synthesis directly within silk protein matrices, including membranes, scaffolds, hydrogels, coatings, etc., create intimate interactions with superior stability and performance characteristics, also ensuring that nanoparticle formation occurs within the protective environment of the protein matrix and resulting in materials with superior stability and bioactivity. Table 1 provides a comprehensive overview of the primary synthesis methods employed for creating these hybrid materials, highlighting the specific conditions, resulting nanoparticle characteristics, and key advantages of each approach.

These revolutionary approaches harness the inherent chemical complexity of silk proteins, which can serve as sophisticated nanomaterial synthesis platforms. The functional carboxylate groups act as a reducing agent for the synthesis of silver nanoparticles, while both NH^2+^ and COO^−^ act as a stabilizer of AgNPs [49].

Recent groundbreaking research by Agudelo and colleagues has systematically demonstrated how the concentration of the precursor salt and the reducing agent, along with the pH conditions significantly influence the reducing properties of silk fibroin in green silver nanoparticle synthesis [58]. The stabilizing effect of silk fibroin waste (SFw) on silver nanoparticles varies with reaction pH. Higher pH values correspond to increased electrokinetic potential and enhanced stability, particularly above pH 10. At neutral pH, charged acidic and basic groups facilitate electrostatic attractions between oppositely charged species. However, when pH exceeds 9.0, basic group deprotonation begins while fully deprotonated acidic groups create charge repulsion due to reduced electrostatic interactions and the predominant negative charge from carboxylate groups in the protein framework. At pH 11, the electrokinetic potential decreases, likely attributed to dendritic structure formation that alters repulsive forces through particle–particle attractive interactions, resulting in more extensively branched configurations [58]. In Shivananda’s study, colloidal AgNPs were synthesized under incandescent light exposure utilizing an aqueous silk fibroin solution as a green stabilizer and reducing agent. In colloidal chemistry the light is adopted as a tool for the nucleation and growth of metal nanoparticles, with light–particle interaction regulating particle size, shape, and composition [59].

UV-photoreduction methods have emerged as particularly effective approaches for silver reduction in silver treated protein matrices. The process follows fundamental reaction pathway where, on a silk protein substrate, silver nitrate is converted in nanoparticles under UV irradiation. This method offers remarkable advantages in terms of adhesion of the silver coating to the surface of the material and long-term antibacterial efficacy. Scientific studies performed on different silk based substrates have demonstrated that this method allows the simultaneous synthesis and deposition in situ of silver nanoparticles onto the surface of the material without the use of any binder, with important advantages in terms of green methodology including the elimination of toxic reducing agents, the reduction in hazardous waste generation, and the achievement of environmentally sustainable nanoparticle synthesis that aligns with principles of green chemistry and eco-friendly manufacturing processes [23,26,60,61]. Silk fibroin is also used as a biotemplate to produce silver nanoparticles in situ under both incandescent light and sunlight at room temperature, serving as the reducing agent of silver, the dispersing and stabilizing agent of the resulting silver nanoparticles [18]. Silk fibroin is made up of 5263 amino acids residues composed of tyrosine, alanine, valine, serine, glycine. The tyrosine residues in silk have potent electron donating capabilities, which presents silk as an attractive template for AgNP biosynthesis as a reducing as well as stabilizing agent [61]. The investigation by Patil et al. presents the development of silk fibroin-based tissue engineering scaffolds incorporating silver nanoparticles that maintain superior antimicrobial characteristics while preserving essential cytocompatibility and stem cell differentiation capabilities. Their experimental approach utilized in situ synthesis methodology, employing silk fibroin simultaneously as both reducing and stabilizing agent for nanoparticle formation [61]. Aramwit et al. selected silk sericin as a reducing and a stabilizing agent for the synthesis of AgNPs under an alkaline condition and evaluated the effects of pH and concentrations of AgNO_3_ and SS on the formation of SS-capped AgNPs. The authors found that, at pH 11, all concentrations of SS and AgNO_3_ formed SS-capped AgNPs with different yields, because of the availability of functional carboxylate groups with reducing potential obtained from the alkaline degradation. Furthermore, the presence of COO^−^ and NH_2_^+^ groups stabilized the AgNPs and prevented their precipitation or aggregation [62,63].

Advanced synthesis strategies continue to evolve, enabling optimization protocols of both nanoparticle properties and protein functionalization into sophisticated hybrid materials with precisely controlled characteristic and unprecedented features over synthesis conditions. The development of AgNP–silk protein nanocomposites requires careful consideration of synthesis methodology, as different approaches yield materials with distinct properties and performance characteristics. A critical consideration for clinical translation and long-term safety profiles is represented by the biodegradability and clearance mechanisms of AgNP–silk protein nanocomposites. Silk proteins undergo controlled enzymatic degradation by proteolytic enzymes such as protease XIV, with degradation rates ranging from weeks to months depending on processing conditions and the secondary structure of the silk, particularly its β-sheet content [64].

When silver nanoparticles are embedded within a silk matrix, the structural features that govern silk degradation also modulate silver release, with the β-sheet content significantly influencing AgNP release profiles in vitro and in vivo [65]. Complementary in vivo studies have demonstrated that silver is progressively cleared from most organs after various exposure routes [66].

Together, these findings indicate that silk fibroin–AgNP composites integrate tunable scaffold resorption with physiologically compatible silver clearance, thereby minimizing long-term tissue accumulation and supporting their safe clinical translation.

Moreover, the integration of silk proteins and silver nanoparticles with complementary materials creates synergistic hybrid systems that exhibit enhanced properties beyond individual components. For example, poly(vinyl alcohol) (PVA) blended with silk fibroin–silver nanocomposites demonstrates improved mechanical strength, enhanced antimicrobial activity, and superior processability for electrospinning applications [67]. The incorporation of gelatin into silk fibroin–silver systems creates triple-component nanofibers with exceptional wound healing properties, where gelatin provides additional cell adhesion sites while maintaining the antimicrobial efficacy of silver nanoparticles [68]. Chitosan–silk fibroin–silver nanocomposites exhibit remarkable pH-responsive behavior, enabling controlled drug release in specific physiological environments while providing broad-spectrum antimicrobial protection [55]. The integration with poly(lactic-co-glycolic acid) (PLGA) creates biodegradable drug delivery systems with tunable degradation rates and sustained antimicrobial activity [69].

These multi-component systems demonstrate the versatility of silk–silver nanocomposites as foundational materials for complex biomedical applications requiring multiple functionalities in a single platform [70].

## 5. Synergistic Antimicrobial Mechanisms and Performance Enhancement for Biomedical Applications

The integration of silver nanoparticles with silk proteins creates sophisticated antimicrobial systems where molecular-level interactions generate remarkable emergent properties that remain unattainable by individual components operating independently. This synergy operates through interconnected mechanisms that collectively enhance therapeutic efficacy while simultaneously minimizing adverse effects, representing a fundamental advancement in antimicrobial material design. The development of responsive release systems that adapt to local tissue conditions represents intelligent options to modulate their antimicrobial activity based on real-time assessment of local infection status, inflammatory markers, and tissue healing progress. Such adaptive systems promise to optimize therapeutic outcomes while minimizing adverse effects, representing a technological evolution from static antimicrobial agents to dynamic, responsive therapeutic platforms. The antimicrobial efficacy of AgNP–silk protein nanocomposites emerges from sophisticated molecular interactions that create synergistic effects beyond the capabilities of individual components. To provide a systematic understanding of these complex mechanisms, Table 2 summarizes the primary antimicrobial pathways, the specific roles of silver and silk proteins, and their practical applications in biomedical contexts.

Silk proteins enable remarkably sophisticated release control mechanisms that respond dynamically to local tissue conditions. Lin et al. focused on the construction and functional characterization of different SF-based delivery systems, mainly including various exogenous and endogenous stimuli including temperature, light, magnetic field, pH, and enzyme responses. In response to external conditions such as pH, the secondary structure and conformation of SF can change. Low pH can enhance interchain aggregation due to highly hydrophilic groups, whereas at high pH, the repulsion of charged carboxyl groups occurs [76]. Enzymatic triggering also represents another silk protein-mediated release control, for creating an intelligent therapeutic system with enhanced antimicrobial activity and minimized potential side effects [76].

**Figure 2:**
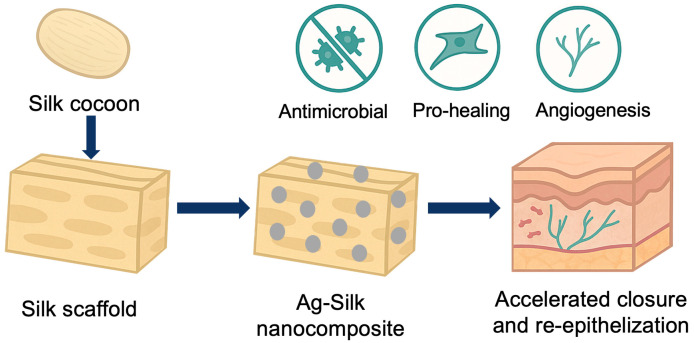
Representation of an integrated Ag–silk system for wound healing application, where the synergistic action of silver nanoparticles and silk matrix contribute simultaneously to infection control and tissue regeneration.

Silk protein integration offers a natural solution for providing biocompatible matrices that can be tailored for specific clinical applications while maintaining the fundamental antimicrobial advantages that make silver nanoparticles such promising therapeutic agents for a wide range of biomedical applications. The versatility of AgNP–silk protein nanocomposites extends across multiple biomedical applications, each leveraging specific aspects of the synergistic antimicrobial and regenerative properties. Figure 2 shows representative examples of integrated systems, showcasing how these materials can be tailored for diverse therapeutic contexts including wound healing, infection control, and tissue engineering applications.

Arumugan et al. designed and developed silver and gold nanoparticles incorporated into silk fibroin and gelatin composite nanofibers through the electrospun method for wound healing applications. Their results demonstrated that SF/GL/Ag-Au composite nanofiber scaffolds significantly accelerated wound closure, achieving a remarkable 99.58% wound healing rate in the 21 days compared to control groups [68].

Suaza et al. prepared PVA/Bombyx mori fibroin/silver nanoparticles (Ag-NPs) composite nanofiber scaffolds by electrospinning and investigated the effect of fibroin and silver nanoparticles on the PVA membranes, finding an increase in the stiffness. Moreover, biological tests demonstrated that at 36 h of culture the number of MG-63 cells seeded on the PVA18% wt/SF/Ag-NPs scaffolds exceeded the control, and that at 10 days of culture scaffolds’ mineralization increased in comparison with pristine membrane and the control, indicating early osteogenic differentiation [67]. Shao et al. successfully fabricated unilateral silver-loaded silk fibroin difunctional membranes as antibacterial wound dressings through a simplified layer-by-layer technique. Both sides of the Ag-SF/SF membrane exerted an antibacterial effect, and the SF side promoted wound healing efficiently. While exhibiting strong antibacterial properties, studies on cell viability revealed good cytocompatibility and suggested improved expression levels of Col I and TGF-β mRNA in vitro [71]. The silver/fibroin composite nanofibers developed by Khan et al. through electrospinning demonstrated a role of silver in increasing the charge density in polymer solution and in creating thinner fibers compared to pristine ones. The addition of silver also restrains the growth of any microorganism, thus providing the desired antimicrobial activity to the SF nanofiber for its employment in tissue engineering [77].

Systematic studies have demonstrated remarkable performance improvements when silver nanoparticles and silk proteins are synergistically combined. Tahir et al. demonstrated that sericin-conjugated silver NPs significantly inhibit the growth of *E. coli*, *S. aureus*, and *K. pneumoniae*. The authors synthesized silver NPs by adding different concentrations of silver nitrate (0.5–30 mg) in 0.2% sericin, followed by and exposure to natural light. The NPs were synthesized using sericin as a reducing agent and resulted stable at different temperatures and pH [78]. To improve the anti-bacterial performance of porous silkworm cocoon-based wound films, Yu et al. have incorporated the silk sericin as a reducing agent for the conversion of Ag^+^ to Ag. The wound dressings exhibited excellent biocompatibility, anti-bacterial performance, good extensibility and significantly accelerated the healing rate in vivo in infected wounds in New Zealand White rabbits [79]. The in vivo studies performed by Gok et al. aimed to evaluate the performance of silver nanoparticles coated with sericin adsorbed on poly (ethylene terephthalate)-g-poly(hydroxyethylmethacrylate) nanofibers. The healing process of deep burn wounds in Sprague-Dawley male rats was monitored for 21 days and the produced nanofibers were more effective in wound healing than the control group. It was found that the nanofibers modified with sericin and silver nanoparticles had antimicrobial effects on both Gram-positive and Gram-negative bacteria [80].

Excellent antibacterial activity and biofilm-inhibition ability against *S. aureus* and *P. aeruginosa* were exhibited by the AgNPs embedded silk sericin-based sponges developed by Tao et al. The biomimetic dressings were obtained by blending sericin with PVA and AgNO_3_ through repetitive freeze-thawing, exploiting the redox property of Tyr for synthesizing the silver nanoparticles. Through an environment-friendly process, the materials were proposed to efficiently accelerate tissue restoration, angiogenesis, and collagen deposition to promote wound healing [81]. Indeed, fibroin has been recognized as effective in stimulating cell growth and migration, promoting pro-angiogenic action, and significantly accelerating skin wound healing through multiple signaling pathways [82].

Silk sericin has been shown to modulate inflammatory responses by inhibiting pro-inflammatory cytokines, thus reducing inflammation and promoting the environment for healing. Furthermore, the antioxidant properties of sericin help to neutralize free radicals in wounds and, mitigating oxidative stress, SS helps protect the wound tissue supporting the natural healing process. In addition to the anti-inflammatory and antioxidant activities, SS exhibits antibacterial properties [83]. Wang et al. reported that the antibacterial activity of sericin may be attributed to the cationic nature, which interacts with negatively charged bacterial cell membranes, leading to altered permeability and inducing protein leakage and other intracellular components of the bacteria. Moreover, the antibacterial activity of sericin may be related to the presence of cysteine with sulfhydryl group and to other small components, including protease inhibitors and seroins [84].

On the other hand, ability to modulate inflammatory responses while promoting angiogenesis has been attributed to silver. Enhanced fibroblast migration has been observed in silver-treated scaffolds, which could be related to the upregulation of wound healing-associated genes such as VEGF and TGF-β1, which, respectively, promote angiogenesis and fibroblast chemotaxis, and regulate fibroblast proliferation, collagen synthesis, and epithelial–mesenchymal transition [26]. In murine burn wound models, topical AgNP treatment was shown to reduce early neutrophil infiltration and to down-regulate pro-inflammatory cytokines such as interleukin-6 (IL-6) and tumor necrosis factor-α (TNF-α), thereby limiting excessive inflammation and accelerating re-epithelialization and granulation tissue formation [85]. In vivo data further demonstrate that AgNPs promote macrophage polarization from classically activated M1 to alternatively activated M2 phenotypes, an essential step for the resolution of inflammation and for stimulating angiogenesis and extracellular matrix deposition [86,87]. These immunomodulatory actions complement the well-established antibacterial activity of silver, providing a dual mechanism through which AgNP-containing biomaterials enhance wound healing. Table 3 provides an overview of promising results achieved in wound healing application, where biological properties relate to the materials developed, the AgNPs size and the silk form.

This comprehensive integration of antimicrobial activity, biocompatibility enhancement, and tissue regeneration promotion represents a paradigmatic advancement in therapeutic material design, where synergistic effects create emergent properties that fundamentally exceed the capabilities of individual components.

The extensive validation through controlled studies provides a robust foundation for clinical translation and represents a valuable approach to address the complex challenges of infection control and tissue repair in modern healthcare. Although evidence converges on multi-mechanistic efficacy, cross-study comparability remains hampered by heterogeneous assays, variable antibacterial testing protocols, and limited long-term stability data under physiologically relevant flow/enzymatic conditions; these gaps motivate future works on this relevant topic.

## 6. General Discussion and Conclusions

**Figure 3:**
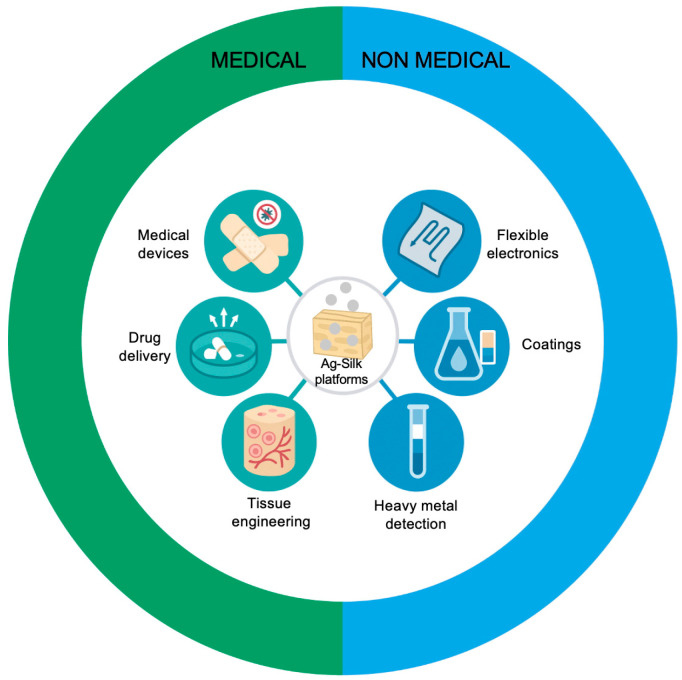
Medical and non-medical applications of AgNP–silk protein nanocomposites, evidencing the technological potential of these biomimetic materials.

This narrative review has been conceived as a critical discussion on the current literature on silver–silk protein nanocomposites, presenting recent experimental results achieved by different research groups. Existing evidence is integrated and compared to highlight converging findings, unresolved gaps, and future research needs in this emerging field. The following concluding remarks summarize the key concepts and perspectives that arise from this integrated analysis. Building on these considerations, it is clear that the unique molecular architecture and multifunctionality of silk proteins open opportunities that extend well beyond their classical biomedical use. In particular, the intrinsic potential of silk polymers extends far beyond their traditional textile applications, positioning them as promising materials for advanced nanotechnology applications. The unique hierarchical structure of silk proteins, characterized by alternating crystalline β-sheet regions and amorphous domains, enables unprecedented tunability of material properties through controlled processing conditions. Recent advances in silk protein engineering have revealed their capacity for self-assembly into diverse nanostructures including nanofibers, nanospheres, and hydrogels with precisely controlled porosity and surface functionalization. Furthermore, silk proteins exhibit remarkable thermal stability, maintaining structural integrity at temperatures exceeding 200 °C, which expands their applicability in high-performance applications. Beyond biomedical use, silk–Ag conjugates prepared via these chemistries are being explored as functional materials in sensing and flexible devices. Emerging applications of AgNP–silk protein nanocomposites in next-generation technologies include flexible electronics, environmental sensing, and smart material systems, demonstrating the broader technological potential of these biomimetic materials (Figure 3).

Silk’s optical transparency and mechanical flexibility with silver’s superior electrical conductivity can create transparent conductive films suitable for flexible electronics. Other examples including heavy metal detection and highly sensitive colorimetric sensors also open new scenarios towards advanced applications of silk protein–silver nanoparticles nanocomposites [91].

The biodegradability of silk proteins can be precisely controlled through crosslinking strategies and molecular weight modulation, enabling sustained release applications and temporary implant technologies.

The synergistic integration of silk proteins with silver nanoparticles represents a significant advancement in antimicrobial biomaterial design that transcends conventional therapeutic limitations. This review has demonstrated that AgNP–silk protein nanocomposites constitute a transformative platform technology capable of addressing multifaceted challenges of antimicrobial resistance while promoting tissue regeneration. This biomimetic approach exemplifies nature’s strategies for achieving optimal performance through sophisticated integration of multiple functional elements at different hierarchical scales, creating emergent properties that exceed individual component capabilities.

The evidence presented establishes several key scientific contributions. First, silk proteins function as sophisticated biological nanofactories that enable environmentally sustainable AgNP synthesis while serving as intelligent delivery matrices with stimulus-responsive capabilities. The natural reducing, stabilizing, and capping properties of both fibroin and sericin eliminate the need for toxic chemicals traditionally required in nanoparticle synthesis, creating a green nanotechnology approach that aligns with sustainability principles. Second, the synergistic mechanisms operate through multiple interconnected pathways that collectively enhance therapeutic efficacy far beyond simple additive effects. The pH-responsive release systems, enzymatic triggering mechanisms, and multi-target bacterial disruption strategies create intelligent therapeutic platforms that adapt dynamically to local physiological conditions, providing enhanced antimicrobial activity precisely where and when needed. Most significantly, clinical applications demonstrate remarkable wound healing acceleration, with composite systems achieving improved wound closure rates within 21 days while maintaining excellent cytocompatibility and promoting tissue regeneration. This integration creates emergent properties that fundamentally exceed the capabilities of individual components, establishing a new paradigm for therapeutic material design that works in harmony with biological systems.

At the same time, some current limitations and research gaps that still must be addressed need to be highlighted to fully realize these opportunities. The literature shows highly heterogeneous synthesis protocols and antibacterial testing methods, which makes cross-study comparison and meta-analysis difficult. Only a limited number of investigations report long-term stability or silver release kinetics under physiologically relevant conditions, and standardized characterization criteria still require more attention. Coordinated efforts toward harmonized protocols, reproducibility validation, and scalable production technologies will therefore be crucial to support clinical and technological deployment of AgNP–silk protein nanocomposites.

In conclusion, silver–silk protein nanocomposites constitute a next-generation biomimetic platform that combines green synthesis, multi-target antimicrobial activity, and tissue-regenerative capacity. Their unique integration of biocompatibility, tunable degradation, and responsive silver release positions these materials as promising candidates for infection control and advanced wound healing. Consolidating standardized synthesis and characterization protocols will be critical to translate these scientific advances into clinical and industrial practice.

## 7. Future Perspectives

The integration of AgNP–silk protein nanocomposites with other biomaterials and the development of multifunctional hybrid systems represent promising avenues for next-generation biomedical applications. As the field continues to evolve, the synergistic combination offers a pathway toward achieving dual goals of effective infection control and enhanced tissue healing in intelligent therapeutic platforms.

However, despite these promising developments, several challenges must be addressed to fully realize the clinical potential of AgNP–silk protein nanocomposites. Standardization of synthesis protocols, comprehensive long-term biocompatibility assessments, and scalable manufacturing processes represent critical areas requiring continued research attention. Additionally, the development of predictive models for optimizing nanocomposite properties based on specific clinical applications are essential for translating laboratory successes into clinical realities. Furthermore, the development of smart responsive systems that can adapt their therapeutic profiles based on real-time biological feedback represents the next frontier in intelligent antimicrobial therapy.

A critical examination of the current literature reveals significant concerns regarding reproducibility and validation in AgNP–silk protein research. Despite promising initial results, systematic replication studies that validate previously reported synthesis protocols and therapeutic outcomes remain scarce. Most published works represent proof-of-concept investigations rather than validation studies, with limited standardization of experimental conditions, characterization methods, and biological assessment protocols. Indeed, the absence of standardized characterization protocols makes it difficult to compare results across different research groups, hindering the establishment of structure–activity relationships essential for rational material design. Addressing these reproducibility challenges requires coordinated efforts to develop standardized protocols for synthesis, characterization, and biological evaluation, along with systematic validation studies that confirm the reliability and scalability of the most promising approaches reported in the literature. Translational progress will benefit from protocol standardization for Ag^+^ release and ROS metrics, rigorous assessment of long-term stability/biodegradation and cytotoxicity. Future developments should focus on standardization of synthesis protocols and comprehensive biocompatibility assessments, exploration of alternative silk protein sources and advanced manufacturing technologies including 3D bioprinting and microfluidic synthesis, development of smart responsive systems adapting therapeutic profiles based on real-time biological feedback, and expansion into emerging applications including wearable sensors, flexible electronics, and environmental monitoring systems.

## Figures and Tables

**Table 1:** Examples of synthesis methods for AgNP–silk nanocomposites.

Synthesis Method	Typical Conditions	Silk Form	AgNP Size	Key Features	Refs.
In situ reduction	AgNO_3_ + silk solution, green chemistry	Films/fibers/scaffolds	5.66–14 nm	Simple; biocompatible; green	[10,50,51]
Photo-assisted reduction	UV lamp + aqueous solutions	Coatings/films/hydrogel	100–150 nm	Enhanced adhesion; long-term efficacy	[52,53]
Electrospinning + precursors	SF electrospinning with AgNP precursors	Nanofibers	not specified	Scalable, porous fibers	[54,55]
Green synthesis via sericin	Sericin-mediated reduction in aqueous solution	Colloids/coatings	~20–120 nm	Eco-friendly; bioactive corona	[15,56,57]
Lyophilization (freeze-drying)	Incorporate AgNPs into porous NP–silk foams	3D porous foams	~100 nm	High porosity; high adhesion and NP distribution	[6,23,26]

**Table 2:** Main antibacterial mechanisms of AgNP–silk nanocomposites.

Mechanism	Role of Ag	Role of Silk Proteins	Developed Materials	Application	Refs
Controlled Ag^+^ release from silk matrix	Local Ag^+^ release, enzyme inhibition; intracellular damage	Sericin/fibroin reduce/cap AgNP and stabilize them; matrix modulates release	Unilateral SF membrane Ag-loaded; SF scaffold Ag-treated; Electrospun SF fibers with AgNP	Wound dressings/infection control	[23,54,71,72]
ROS-mediated killing from silk-delivered AgNP/Ag^+^	AgNP/Ag^+^ trigger ROS, causing oxidative lesions	Silk acts as reservoir/vehicle, dosing Ag while improving wound healing	Sericin–AgNP (green synthesis) dispersions/gels; Sericin/AgNP hydrogels	Antimicrobial coatings; wound care	[49,56,73]
Membrane disruption by contact + Ag^+^	Surface-bound AgNP/Ag^+^ increase contact-killing and permeabilization	Silk substrates (films/fibers) immobilize/anchor AgNP on the surface	Electrospun SF fibers–AgNP; silk fibroin film with in situ Ag	Wound films, dressings	[52,54]
Biofilm inhibition on silk surfaces	AgNP/Ag^+^ prevent adhesion and disrupt biofilm	Silk provides contact interface and localized release over time	AgNP hydrogel; fibroin–Agsolution/film; silk + AgNP scaffold	Topical therapy; burn/acute wounds; anti-biofilm dressings	[10,74,75]

**Table 3:** Representative silk protein–AgNP composites and their biological and wound healing outcomes.

Study/Material	AgNP Size (nm)	Silk Protein Concentration	Biocompatibility	Antimicrobial Activity	Wound-Healing	Refs.
Sericin/fibroin-capped AgNPs (colloids)	5–30; 6–34; 20–70	Sericin ~0.2, 1.5% *w*/*v*; Fibroin 1.5%–3% *w*/*v*.	>80% viability; in vivo negligible hemolysis	MIC values 20 μg/mL (*E. coli* and *P. aeruginosa*); 7.81 μg/mL (*Pseudomonas* sp.); 25–100 µg/mL (*E. coli*, *Enterococcus faecium*, *Listeria monocytogenes*); 11–26 mm inhibition zone (*E. coli*, *S. aureus*, *K. pneumoniae*)	Scratch assay: 64–67.7% closure	[32,49,88]
Silk scaffold with AgNPs	5.66–8.82	solid cocoon, scaffold, fibroin scaffolds	Hemocompatible; cell viability > 80%	inhibition zone 5.5 mm (*P. aeruginosa*), 95% reduction for *Escherichia coli* and 92% for antibiotic-resistant *Pseudomonas aeruginosa*	100% wound closure in vitro	[10,23,26,89]
SF-Ag based hydrogels	23; 40; 92	5% (*w*/*v*) silk sericin; 5% (*w*/*v*) lk fibroin	Cell viability > 70–80%	Broad spectrum antibacterial activity; inhibition zone larger than 1 mm (*E. coli* and *S. aureus*)	92.74% wound healing in vivo	[62,78,90,91]
SF-Ag fibers	10; 220	Fibroin fibers	Cell viability 90%	MIC values 125 μg mL (*E. coli*); bacterial reduction 99.2% (*S. aureus*) and 98.0% (*E. coli*)	99.58% wound healing in vivo	[54,61,68,71]

## References

[1] GBD 2021 Antimicrobial Resistance Collaborators (2024). Global burden of bacterial antimicrobial resistance 1990–2021: A systematic analysis with forecasts to 2050. Lancet.

[2] Galgano M., Pellegrini F., Catalano E., Capozzi L., Del Sambro L., Sposato A., Lucente M.S., Vasinioti V.I., Catella C., Odigie A.E. (2025). Acquired Bacterial Resistance to Antibiotics and Resistance Genes: From Past to Future. Antibiotics.

[3] Duman H., Eker F., Akdaşçi E., Witkowska A.M., Bechelany M., Karav S. (2024). Silver Nanoparticles: A Comprehensive Review of Synthesis Methods and Chemical and Physical Properties. Nanomaterials.

[4] Kumar L., Bisen M., Harjai K., Chhibber S., Azizov S., Lalhlenmawia H., Kumar D. (2023). Advances in Nanotechnology for Biofilm Inhibition. ACS Omega.

[5] Qing Y., Cheng L., Li R., Liu G., Zhang Y., Tang X., Wang J., Liu H., Qin Y. (2018). Potential antibacterial mechanism of silver nanoparticles and the optimization of orthopedic implants by advanced modification technologies. Int. J. Nanomed..

[6] Paladini F., Pollini M. (2022). Novel Approaches and Biomaterials for Bone Tissue Engineering: A Focus on Silk Fibroin. Materials.

[7] Panico A., Paladini F., Pollini M. (2019). Development of regenerative and flexible fibroin-based wound dressings. J. Biomed. Mater. Res. Part B Appl. Biomater..

[8] Schmidt T., Puchalla N., Schendzielorz M., Kramell A.E. (2023). Degumming and characterization of *Bombyx mori* and non-mulberry silks from Saturniidae silkworms. Sci. Rep..

[9] Chartvivatpornchai N., Okahisa Y. (2025). Structural and mechanical comparison of Eri and Mulberry silk fibroin nanofibers films through advanced mechanical treatments for sustainable applications. Int. J. Biol. Macromol..

[10] Dam P., Shaw S., Mondal R., Chakraborty J., Bhattacharjee T., Sen I.K., Manna S., Sadat A., Suin S., Sarkar H. (2024). Multifunctional silver nanoparticle embedded eri silk cocoon scaffolds against burn wounds-associated infection. RSC Adv..

[11] Qi Y., Wang H., Wei K., Yang Y., Zheng R.Y., Kim I.S., Zhang K.Q. (2017). A Review of Structure Construction of Silk Fibroin Biomaterials from Single Structures to Multi-Level Structures. Int. J. Mol. Sci..

[12] Gallo A.L., Pollini M., Paladini F. (2018). A combined approach for the development of novel sutures with antibacterial and regenerative properties: The role of silver and silk sericin functionalization. J. Mater. Sci. Mater. Med..

[13] Paladini F., D’Urso F., Panico A., Lanzillotti C., Broccolo F., Pollini M. (2025). Comparative Analysis of Highly Purified Sericin and Waste-Derived Sericin: Implications for Biomedical Applications. Biomimetics.

[14] Reizabal A., Costa C.M., Pérez-Álvarez L., Vilas-Vilela J.L., Lanceros-Méndez S. (2023). Silk Fibroin as Sustainable Advanced Material: Material Properties and Characteristics, Processing, and Applications. Adv. Funct. Mater..

[15] Shaw S., Mondal R., Dam P., Mandal A., Acharya R., Manna S., Gangopadhyay D., Mandal A.K. (2024). Synthesis, characterization and application of silk sericin-based silver nanocomposites for antibacterial and food coating solutions. RSC Adv..

[16] Guo C., Li C., Kaplan D.L. (2020). Enzymatic Degradation of *Bombyx mori* Silk Materials: A Review. Biomacromolecules.

[17] Belda Marín C., Fitzpatrick V., Kaplan D.L., Landoulsi J., Guénin E., Egles C. (2020). Silk Polymers and Nanoparticles: A Powerful Combination for the Design of Versatile Biomaterials. Front. Chem..

[18] Fei X., Jia M., Du X., Yang Y., Zhang R., Shao Z., Zhao X., Chen X. (2013). Green synthesis of silk fibroin-silver nanoparticle composites with effective antibacterial and biofilm-disrupting properties. Biomacromolecules.

[19] Zafar M.S., Belton D.J., Hanby B., Kaplan D.L., Perry C.C. (2015). Functional material features of *Bombyx mori* silk light versus heavy chain proteins. Biomacromolecules.

[20] Välisalmi T., Linder M.B. (2024). The ratio of fibroin to sericin in the middle silk gland of *Bombyx mori* and its correlation with the extensional behavior of the silk dope. Protein Sci..

[21] Asakura T. (2021). Structure of Silk I (*Bombyx mori* Silk Fibroin before Spinning) -Type II β-Turn, Not α-Helix-. Molecules.

[22] De Giorgio G., Matera B., Vurro D., Manfredi E., Galstyan V., Tarabella G., Ghezzi B., D’Angelo P. (2024). Silk Fibroin Materials: Biomedical Applications and Perspectives. Bioengineering.

[23] Paladini F., Russo F., Masi A., Lanzillotti C., Sannino A., Pollini M. (2024). Silver-Treated Silk Fibroin Scaffolds for Prevention of Critical Wound Infections. Biomimetics.

[24] Puerta M., Peresin M.S., Restrepo-Osorio A. (2020). Effects of Chemical Post-treatments on Structural and Physicochemical Properties of Silk Fibroin Films Obtained From Silk Fibrous Waste. Front. Bioeng. Biotechnol..

[25] Poggi G., Chelazzi D., Laurati M. (2022). Mechanical response and yielding transition of silk-fibroin and silk-fibroin/cellulose nanocrystals composite gels. Colloids Surf. A.

[26] Paladini F., Lanzillotti C., Panico A., Pollini M. (2025). Biological Evaluation of Silver-Treated Silk Fibroin Scaffolds for Application as Antibacterial and Regenerative Wound Dressings. Nanomaterials.

[27] Wang Z., Zhang Y., Zhang J., Luo H., Yao J., Chen X. (2014). Exploring natural silk protein sericin for regenerative medicine: An injectable, photoluminescent, cell-adhesive 3D hydrogel. Sci. Rep..

[28] Wang Y., Gao J., Yang Y., Zhu L., Yang W., Li P., Yang W. (2025). A Review on the Extraction Methods, Bioactivities, and Application in Foods of Silk Sericin. J. Food Biochem..

[29] Seo S.J., Das G., Shin H.S., Patra J.K. (2023). Silk Sericin Protein Materials: Characteristics and Applications in Food-Sector Industries. Int. J. Mol. Sci..

[30] Du Z., Yan Z., Guo Y., Ye R., Tehoungue A., Li Y., Zhang G., Zhang Y. (2025). A high stretchable, transparent sericin hydrogel with antioxidant activity formed by CaCl_2_-formic acid dissolution system. Polymer.

[31] Aad R., Dragojlov I., Vesentini S. (2024). Sericin Protein: Structure, Properties, and Applications. J. Funct. Biomater..

[32] Das G., Shin H.S., Yang I.J., Nguyen L.T.H., Patra J.K. (2025). Silk Biowaste Protein Mediated Silver Nanoparticles Synthesis and Analysis of Anti-Inflammatory, Wound Healing, Antidiabetic, Antioxidant, Tyrosinase Inhibition, and Antibacterial Mechanism of Action. Int. J. Nanomed..

[33] Ellis Tobin S., Brenner S. (2021). Nanotechnology Fundamentals Applied to Clinical Infectious Diseases and Public Health. Open Forum Infect. Dis..

[34] Rodrigues A.S., Batista J.G.S., Rodrigues M.Á.V., Thipe V.C., Minarini L.A.R., Lopes P.S., Lugão A.B. (2024). Advances in silver nanoparticles: A comprehensive review on their potential as antimicrobial agents and their mechanisms of action elucidated by proteomics. Front. Microbiol..

[35] Yin I.X., Zhang J., Zhao I.S., Mei M.L., Li Q., Chu C.H. (2020). The Antibacterial Mechanism of Silver Nanoparticles and Its Application in Dentistry. Int. J. Nanomed..

[36] Sahoo J., Sarkhel S., Mukherjee N., Jaiswal A. (2022). Nanomaterial-Based Antimicrobial Coating for Biomedical Implants: New Age Solution for Biofilm-Associated Infections. ACS Omega.

[37] More P.R., Pandit S., Filippis A.D., Franci G., Mijakovic I., Galdiero M. (2023). Silver Nanoparticles: Bactericidal and Mechanistic Approach against Drug Resistant Pathogens. Microorganisms.

[38] Mikhailova E.O. (2025). Green Silver Nanoparticles: An Antibacterial Mechanism. Antibiotics.

[39] Bruna T., Maldonado-Bravo F., Jara P., Caro N. (2021). Silver Nanoparticles and Their Antibacterial Applications. Int. J. Mol. Sci..

[40] Rohde M.M., Snyder C.M., Sloop J., Solst S.R., Donati G.L., Spitz D.R., Furdui C.M., Singh R. (2021). The mechanism of cell death induced by silver nanoparticles is distinct from silver cations. Part. Fibre Toxicol..

[41] Xu L., Wang Y.Y., Huang J., Chen C.Y., Wang Z.X., Xie H. (2020). Silver nanoparticles: Synthesis, medical applications and biosafety. Theranostics.

[42] Mammari N., Lamouroux E., Boudier A., Duval R.E. (2022). Current Knowledge on the Oxidative-Stress-Mediated Antimicrobial Properties of Metal-Based Nanoparticles. Microorganisms.

[43] Menichetti A., Mavridi-Printezi A., Mordini D., Montalti M. (2023). Effect of Size, Shape and Surface Functionalization on the Antibacterial Activity of Silver Nanoparticles. J. Funct. Biomater..

[44] Dakal T.C., Kumar A., Majumdar R.S., Yadav V. (2016). Mechanistic Basis of Antimicrobial Actions of Silver Nanoparticles. Front. Microbiol..

[45] Stabryla L.M., Johnston K.A., Diemler N.A., Cooper V.S., Millstone J.E., Haig S.-J., Gilbertson L.M. (2021). Role of bacterial motility in differential resistance mechanisms of silver nanoparticles and silver ions. Nat. Nanotechnol..

[46] Pal S., Tak Y.K., Song J.M. (2007). Does the antibacterial activity of silver nanoparticles depend on the shape of the nanoparticle? A study of the Gram-negative bacterium Escherichia coli. Appl. Environ. Microbiol..

[47] Li J., Rong K., Zhao H., Li F., Lu Z., Chen R. (2013). Highly selective antibacterial activities of silver nanoparticles against Bacillus subtilis. J. Nanosci. Nanotechnol..

[48] Stabryla L.M., Moncure P.J., Millstone J.E., Gilbertson L.M. (2023). Particle-Driven Effects at the Bacteria Interface: A Nanosilver Investigation of Particle Shape and Dose Metric. ACS Appl. Mater. Interfaces.

[49] Al Masud M.A., Shaikh H., Alam M.S., Karim M.M., Momin M.A., Islam M.A., Khan G.M.A. (2021). Green synthesis of silk sericin-embedded silver nanoparticles and their antibacterial application against multidrug-resistant pathogens. J. Genet. Eng. Biotechnol..

[50] Agudelo W., Montoya Y., Garcia-Garcia A., Restrepo-Osorio A., Bustamante J. (2022). Electrochemical and Electroconductive Behavior of Silk Fibroin Electrospun Membrane Coated with Gold or Silver Nanoparticles. Membranes.

[51] Zhang Y., Chen X., Li Y., Bai T., Li C., Jiang L., Liu Y., Sun C., Zhou W. (2021). Biomimetic Inorganic Nanoparticle-Loaded Silk Fibroin-Based Coating with Enhanced Antibacterial and Osteogenic Abilities. ACS Omega.

[52] Liu Y., Fan J., Lv M., She K., Sun J., Lu Q., Han C., Ding S., Zhao S., Wang G. (2021). Photocrosslinking silver nanoparticles–aloe vera–silk fibroin composite hydrogel for treatment of full-thickness cutaneous wounds. Regen. Biomater..

[53] Harisha K.S., Shilpa M., Asha S., Parushuram N., Ranjana R., Sangappa Y., Narayana B. (2019). Synthesis of silver nanoparticles using *Bombyx mori* silk fibroin and antibacterial activity. IOP Conf. Ser. Mater. Sci. Eng..

[54] Li Q., Gong H., Jia X., Wang R., Liu Z., Zhang L., Li J., Jiao T. (2024). Electrospinning Silk-Fibroin-Based Fibrous Membranes with AgNPs for Antimicrobial Application. Polymers.

[55] Heydari Foroushani P., Rahmani E., Alemzadeh I., Vossoughi M., Pourmadadi M., Rahdar A., Díez-Pascual A.M. (2022). Curcumin Sustained Release with a Hybrid Chitosan-Silk Fibroin Nanofiber Containing Silver Nanoparticles as a Novel Highly Efficient Antibacterial Wound Dressing. Nanomaterials.

[56] Munir F., Tahir H.M., Ali S., Ali A., Tehreem A., Zaidi S.D.E.S., Adnan M., Ijaz F. (2023). Characterization and Evaluation of Silk Sericin-Based Hydrogel: A Promising Biomaterial for Efficient Healing of Acute Wounds. ACS Omega.

[57] Mehdi M., Qiu H., Dai B., Qureshi R.F., Hussain S., Yousif M., Gao P., Khatri Z. (2021). Green Synthesis and Incorporation of Sericin Silver Nanoclusters into Electrospun Ultrafine Cellulose Acetate Fibers for Anti-Bacterial Applications. Polymers.

[58] Agudelo W., Montoya Y., Garcia-Garcia A., Bustamante J. (2024). Concentration and pH Influence on the Reducing Property of Silk Fibroin from Silk Fibrous Waste in the Green Synthesis of Silver Nanoparticles. J. Nanotechnol..

[59] Shivananda C.S. (2023). Silk fibroin-based green colloidal silver nanoparticle synthesis and their antibacterial and anticancer properties. Inorg. Chem. Commun..

[60] Gallo A.L., Paladini F., Romano A., Verri T., Quattrini A., Sannino A., Pollini M. (2016). Efficacy of silver coated surgical sutures on bacterial contamination, cellular response and wound healing. Mater. Sci. Eng. C Mater. Biol. Appl..

[61] Patil S., Singh N. (2019). Antibacterial silk fibroin scaffolds with green synthesized silver nanoparticles for osteoblast proliferation and human mesenchymal stem cell differentiation. Colloids Surf. B.

[62] Laomeephol C., Punjataewakupt A., Kanchanasin P., Phongsopitanun W., Ferreira H., Neves N.M., Aramwit P. (2025). Silver Cross-Linking of Silk Sericin-Based Hydrogels for Improved Stability and Broad-Spectrum Antimicrobial Properties. ACS Appl. Bio Mater..

[63] Aramwit P., Bang N., Ratanavaraporn J., Ekgasit S. (2014). Green synthesis of silk sericin-capped silver nanoparticles and their potent anti-bacterial activity. Nanoscale Res. Lett..

[64] Jameson J.F., Pacheco M.O., Butler J.E., Stoppel W.L. (2021). Estimating Kinetic Rate Parameters for Enzymatic Degradation of Lyophilized Silk Fibroin Sponges. Front. Bioeng. Biotechnol..

[65] Zhang W., Zhang T., Yan J., Li Q., Xiong P., Li Y., Cheng Y., Zheng Y. (2020). In vitro and in vivo evaluation of structurally-controlled silk fibroin coatings for orthopedic infection and in-situ osteogenesis. Acta Biomater..

[66] Ferdous Z., Nemmar A. (2020). Health Impact of Silver Nanoparticles: A Review of the Biodistribution and Toxicity Following Various Routes of Exposure. Int. J. Mol. Sci..

[67] Mejía Suaza M.L., Leos Rivera J.C., Rodríguez Padilla M.C., Moncada Acevedo M.E., Ossa Orozco C.P., Zarate Triviño D.G. (2023). Poly(vinyl alcohol)/Silk Fibroin/Ag-NPs Composite Nanofibers as a Substrate for MG-63 Cells’ Growth. Polymers.

[68] Arumugam M., Murugesan B., Balasekar P., Malliappan S.P., Chinnalagu D.K., Chinniah K., Cai Y., Mahalingam S. (2023). Silk fibroin and gelatin composite nanofiber combined with silver and gold nanoparticles for wound healing accelerated by reducing the inflammatory response. Process Biochem..

[69] Chen F., Han J., Guo Z., Mu C., Yu C., Ji Z., Sun L., Wang Y., Wang J. (2023). Antibacterial 3D-Printed Silver Nanoparticle/Poly Lactic-Co-Glycolic Acid (PLGA) Scaffolds for Bone Tissue Engineering. Materials.

[70] Shebl H.R., Soliman R.A., Abdallah O.M. (2025). An in vitro study on the efficacy of nanoparticles and nanocomposites as coating materials on surgical sutures. Sci. Rep..

[71] Shao J., Cui Y., Liang Y., Liu H., Ma B., Ge S. (2021). Unilateral Silver-Loaded Silk Fibroin Difunctional Membranes as Antibacterial Wound Dressings. ACS Omega.

[72] Li Y., Zha X., Xiong X., Zhang Y., Feng Y., Xie H., Zhang L., Jiang Q. (2021). A Promising Wound Dressing from Regenerated Silk Fibroin Sponge with Sustain-ed Release of Silver Nanoparticles. J. Renew. Mater..

[73] He H., Tao G., Wang Y., Cai R., Guo P., Chen L., Zuo H., Zhao P., Xia Q. (2017). In situ green synthesis and characterization of sericin-silver nanoparticle composite with effective antibacterial activity and good biocompatibility. Mater. Sci. Eng. C Mater. Biol. Appl..

[74] Jia M., Chen Z., Guo Y., Chen X., Zhao X. (2017). Efficacy of silk fibroin-nano silver against *Staphylococcus aureus* biofilms in a rabbit model of sinusitis. Int. J. Nanomed..

[75] Yathavan B., Chhibber T., Steinhauff D., Pulsipher A., Alt J.A., Ghandehari H., Jafari P. (2023). Matrix-Mediated Delivery of Silver Nanoparticles for Prevention of *Staphylococcus aureus* and *Pseudomonas aeruginosa* Biofilm Formation in Chronic Rhinosinusitis. Pharmaceutics.

[76] Lin X., Cai L., Cao X., Zhao Y. (2023). Stimuli-responsive silk fibroin for on-demand drug delivery. Smart Med..

[77] Khan R.S., Rather A.H., Wani T.U., Rather S.U., Abdal-hay A., Sheikh F.A. (2022). A comparative review on silk fibroin nanofibers encasing the silver nanoparticles as antimicrobial agents for wound healing applications. Mater. Today Commun..

[78] Tahir M.H., Saleem F., Ali S., Ain Q.U., Fazal A., Summer M., Mushtaq R., Zahid M.T., Liaqat I., Murtaza G. (2020). Synthesis of sericin-conjugated silver nanoparticles and their potential antimicrobial activity. J. Basic Microbiol..

[79] Yu K., Lu F., Li Q., Zou H., Xiao J., Liu B., Zhang J., Liu J., Dai F., Wu D. (2017). In situ assembly of Ag nanoparticles (AgNPs) on porous silkworm cocoon-based wound film: Enhanced antimicrobial and wound healing activity. Sci. Rep..

[80] Gök Z.G., Yiğitoğlu M., Vargel İ., Şahin Y., Alçıgır M.E. (2021). Synthesis, characterization and wound healing ability of PET based nanofiber dressing material coated with silk sericin capped-silver nanoparticles. Mater. Chem. Phys..

[81] Tao G., Cai R., Wang Y., Liu L., Zuo H., Zhao P., Umar A., Mao C., Xia Q., He H. (2019). Bioinspired design of AgNPs embedded silk sericin-based sponges for efficiently combating bacteria and promoting wound healing. Mater. Des..

[82] Mazurek Ł., Szudzik M., Rybka M., Konop M. (2022). Silk Fibroin Biomaterials and Their Beneficial Role in Skin Wound Healing. Biomolecules.

[83] Bogadi S., Malayandi R., Raj P.V., Anandasadagopan S.K., Parvathaneni M., Kundu M.K., Islam M.R., Khan F.S., Tagde P., Mondal T.K. (2024). Silk fibroin and sericin: Multifunctional formulations for treating diabetic wound healing. Eur. Polym. J..

[84] Wang S.L., Zhuo J.J., Fang S.M., Xu W., Yu Q.Y. (2024). Silk Sericin and Its Composite Materials with Antibacterial Properties to Enhance Wound Healing: A Review. Biomolecules.

[85] Zhang K., Lui V.C.H., Chen Y., Lok C.N., Wong K.K.Y. (2020). Delayed application of silver nanoparticles reveals the role of early inflammation in burn wound healing. Sci. Rep..

[86] Joorabloo A., Liu T. (2022). Recent advances in nanomedicines for regulation of macrophages in wound healing. J. Nanobiotechnol..

[87] Dahri M., Rezaeian M., Sadeghzadeh H., Beheshtizadeh N., Sadeghi M.M., Zakerhamidi D., Faraji S.N., Pakdel H., Dahri B., Maleki R. (2025). Nanomaterial-driven macrophage polarization: Emerging strategies for immunomodulation and regenerative medicine. Biomed. Pharmacother..

[88] Srivastava C.M., Purwar R., Gupta A.P. (2019). Enhanced potential of biomimetic, silver nanoparticles functionalized Antheraea mylitta (tasar) silk fibroin nanofibrous mats for skin tissue engineering. Int. J. Biol. Macromol..

[89] de Lartigue C., Belda Marín C., Fitzpatrick V., Esposito A., Casale S., Landoulsi J., Guénin E., Egles C. (2024). Silk foams with metallic nanoparticles as scaffolds for soft tissue regeneration. Int. J. Mol. Sci..

[90] Raho R., Nguyen N.Y., Zhang N., Jiang W., Sannino A., Liu H., Pollini M., Paladini F. (2020). Photo-assisted green synthesis of silver doped silk fibroin/carboxymethyl cellulose nanocomposite hydrogels for biomedical applications. Mater. Sci. Eng. C Mater. Biol. Appl..

[91] Shivananda C.S., Kumar P., Vivek M.V., Madhu S., Lakshmeesha Rao B. (2024). Silver nanoparticles reinforced on silk fibroin/carboxymethylcellulose composite films for electrical applications. Mater. Sci. Eng. B.

